# Correction: Ameliorations in dyslipidemia and atherosclerotic plaque by the inhibition of HMG-CoA reductase and antioxidant potential of phytoconstituents of an aqueous seed extract of *Acacia senegal* (L.) Willd in rabbits

**DOI:** 10.1371/journal.pone.0271854

**Published:** 2022-07-18

**Authors:** Jaykaran Charan, Priyanka Riyad, Heera Ram, Ashok Purohit, Sneha Ambwani, Priya Kashyap, Garima Singh, Abeer Hashem, Elsayed Fathi Abd_Allah, Vijai Kumar Gupta, Ashok Kumar, Anil Panwar

The following information is missing from the Acknowledgments: The authors would like to extend their sincere appreciation to the Researchers Supporting Project Number (RSP-2021/134), King Saud University, Riyadh, Saudi Arabia.
