# Supplementary material for: Ameliorations in dyslipidemia and atherosclerotic plaque by the inhibition of HMG-CoA reductase and antioxidant potential of phytoconstituents of an aqueous seed extract of Acacia senegal (L.) Willd in rabbits
Source: PLoS One. 2022 Mar 3;17(3):e0264646. doi: 10.1371/journal.pone.0264646 (PMC8893677; doi:10.1371/journal.pone.0264646)
Supplement: S1 Fig — A: QTOF analyses of aqueous extract of Acacia. senegal (L.) Willd. seed extract. B: QTOF analyses of aqueous extract of Acacia. senegal (L.) Willd. seed extract. C: QTOF analyses of aqueous extract of Acacia. senegal (L.) Willd. seed extract. D: QTOF analyses of aqueous extract of Acacia. senegal (L.) Willd. seed extract. (DOCX) [file pone.0264646.s001.docx]

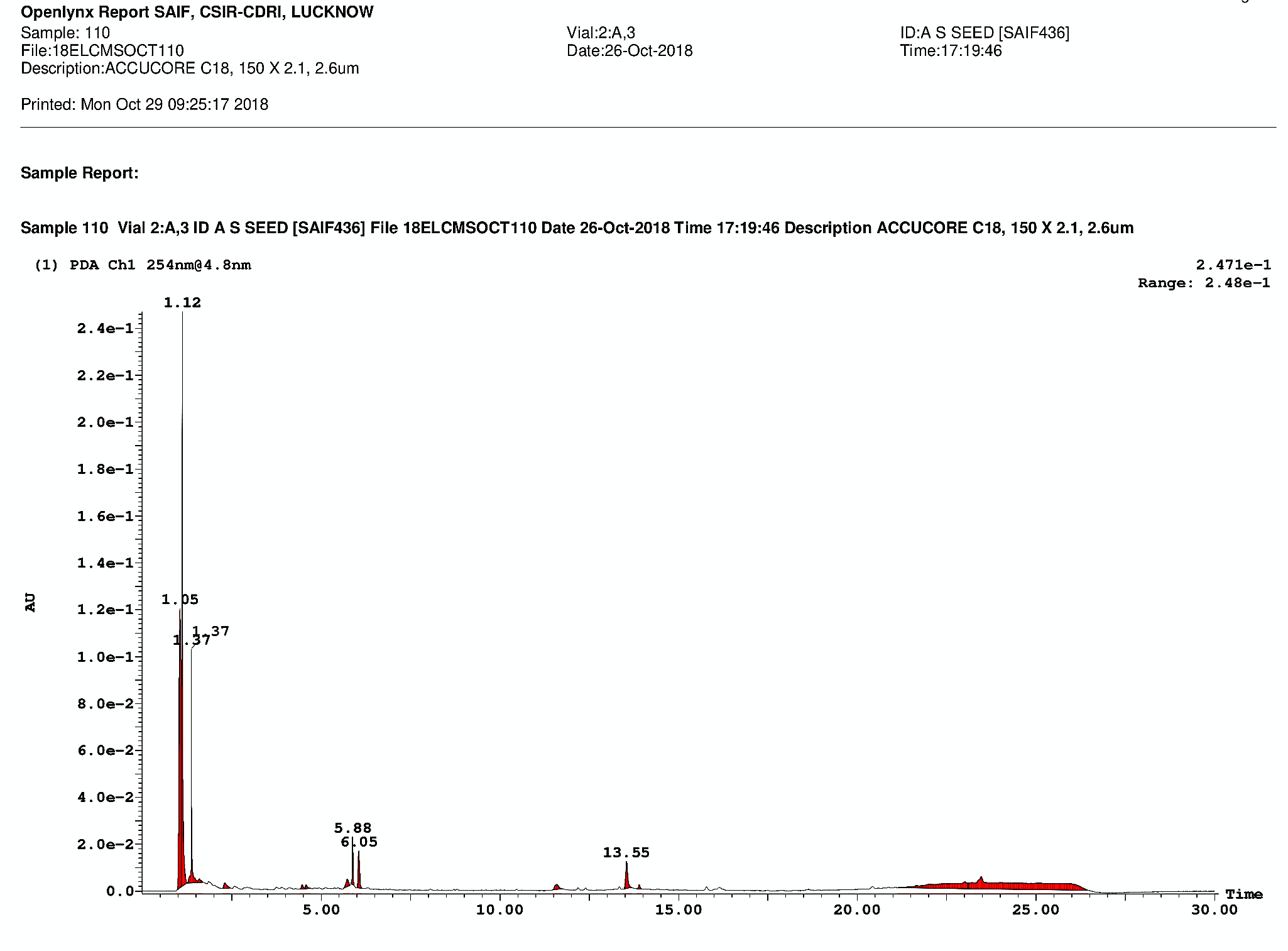


**S1A Fig: QTOF analyses of aqueous extract of *Acacia. senegal* (L.) Willd. seed extract**


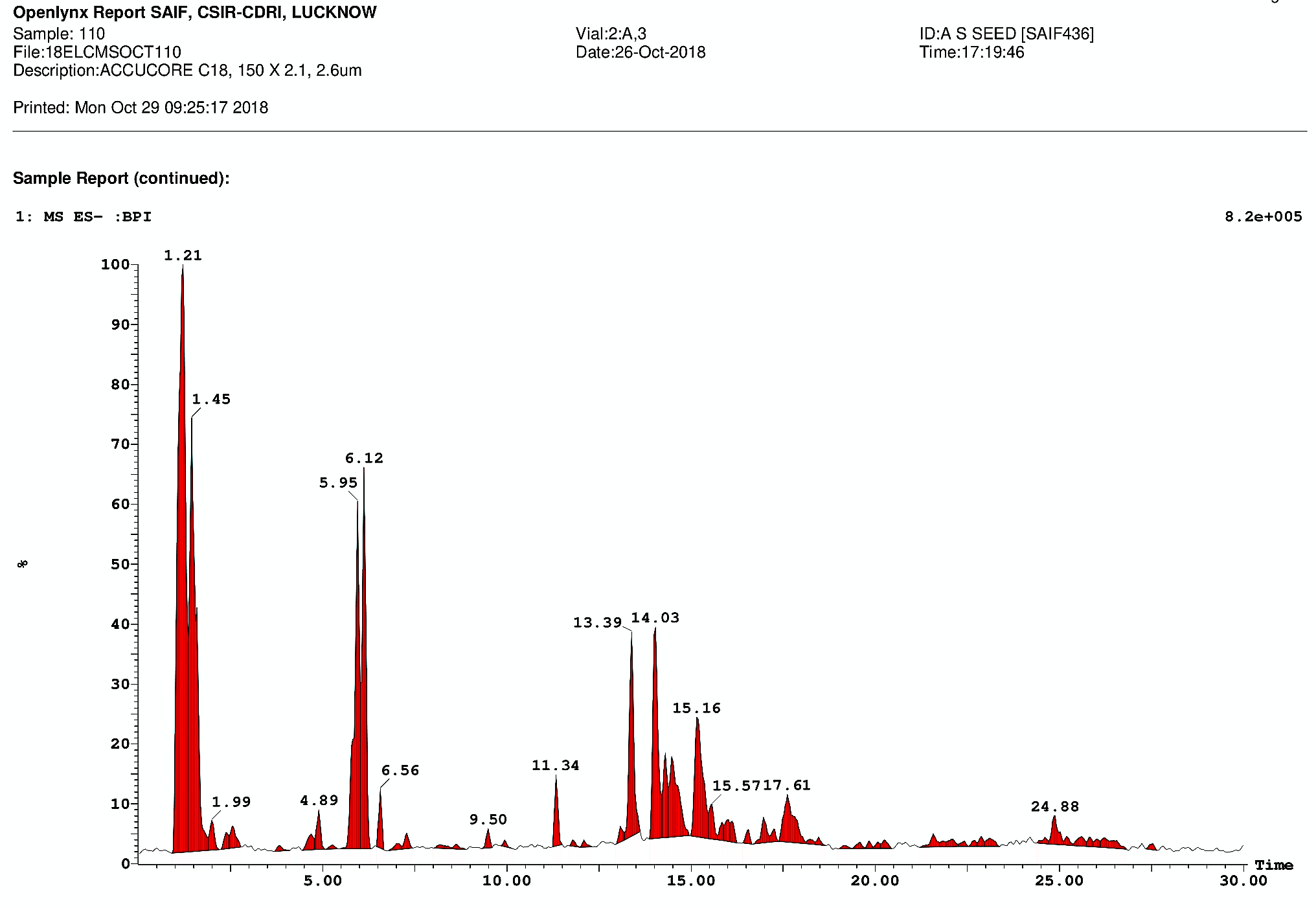


**S1B Fig: QTOF analyses of aqueous extract of *Acacia. senegal* (L.) Willd. seed extract**


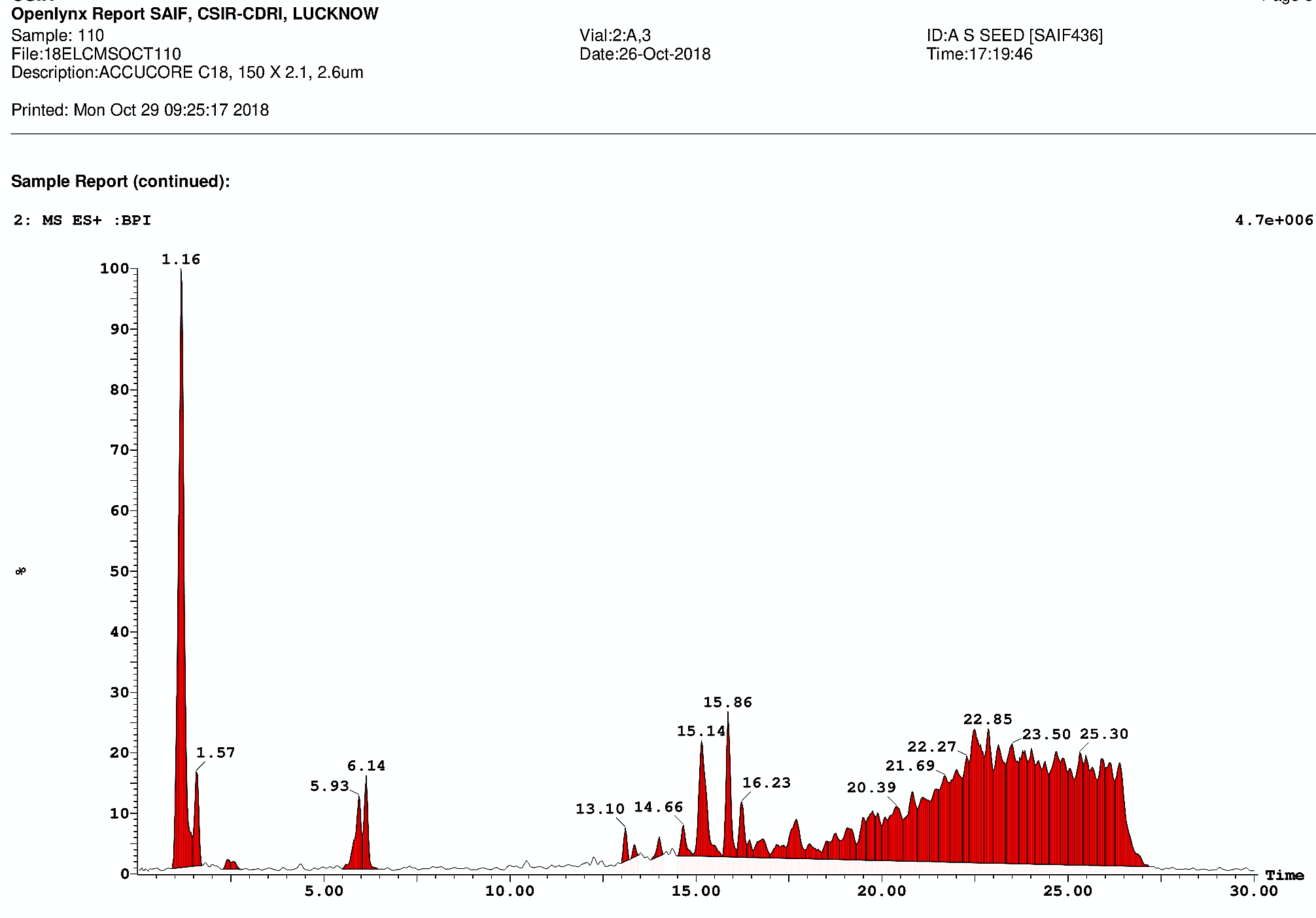


**S1C Fig: QTOF analyses of aqueous extract of *Acacia. senegal* (L.) Willd. seed extract**


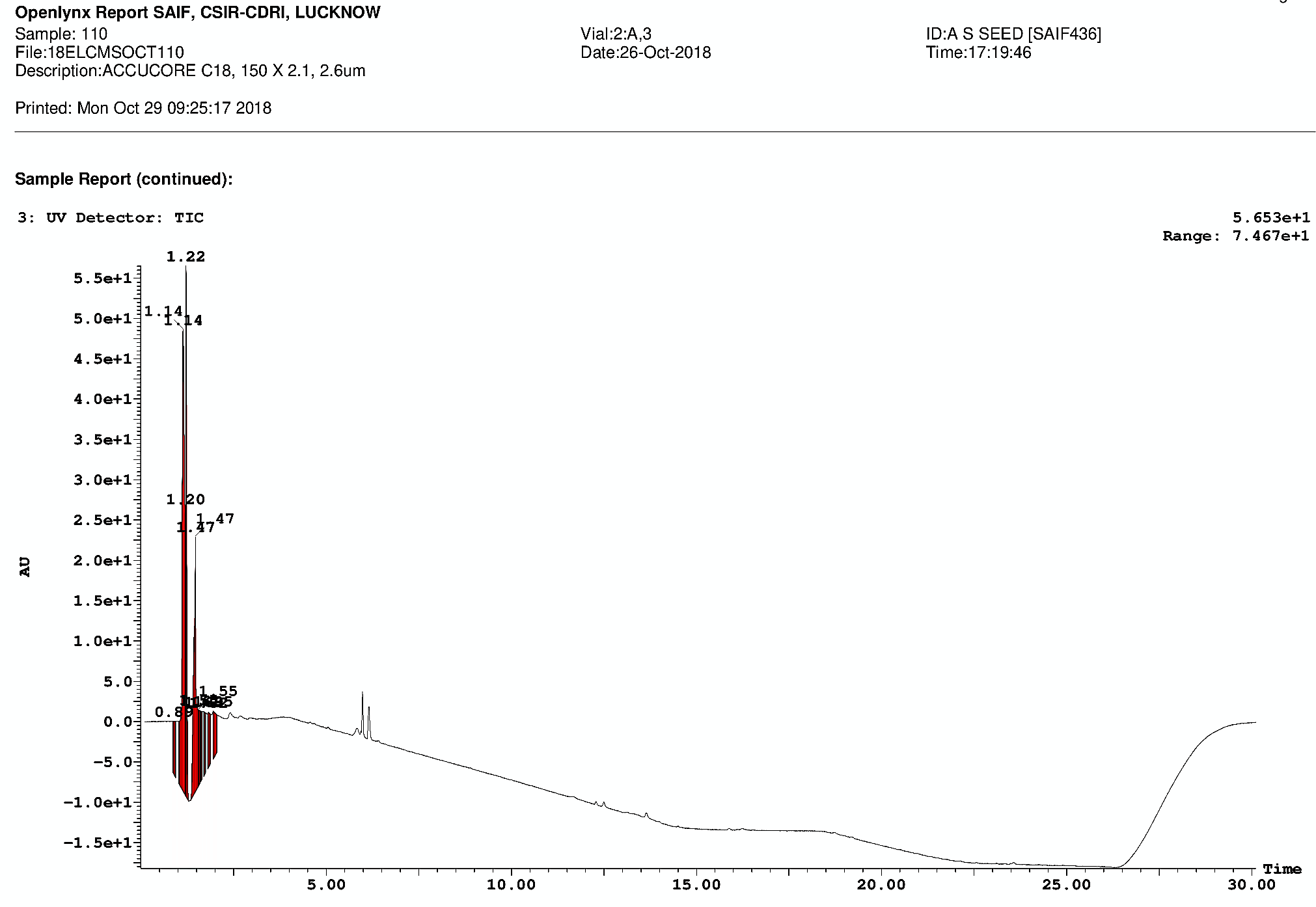


**S1D Fig: QTOF analyses of aqueous extract of *Acacia. senegal* (L.) Willd. seed extract**
