# Supplementary material for: Ameliorations in dyslipidemia and atherosclerotic plaque by the inhibition of HMG-CoA reductase and antioxidant potential of phytoconstituents of an aqueous seed extract of Acacia senegal (L.) Willd in rabbits
Source: PLoS One. 2022 Mar 3;17(3):e0264646. doi: 10.1371/journal.pone.0264646 (PMC8893677; doi:10.1371/journal.pone.0264646)
Supplement: S2 Fig — A: System Temperature graphs of Eicosonoid, flavan-3-ol, linoleic acid, atorvastatin and pravastatin after temperature minimization for 100 picoseconds. B: System Pressure graphs of Eicosonoid, flavan-3-ol, atorvastatin, pravastatin and comparative view accounted after NPT Equilibration for 100ps. C: System Density graphs of linoleic acid, Eicosonoid, flavan-3-ol, atorvastatin, pravastatin and comparative view accounted after Equilibration for 100ps. D: System RMSD of Eicosonoid, Flavan-3-ol, linoleic acid, Pravastatin and atorvastatin. Crystal Backbone (black) Equilibrated Structure Backbone (red). E: Radius of gyration for Eicosonoid, Flavan-3-ol, linoleic acid, atorvastatin and pravastatin system accounted after 1000 ps. (DOCX) [file pone.0264646.s002.docx]

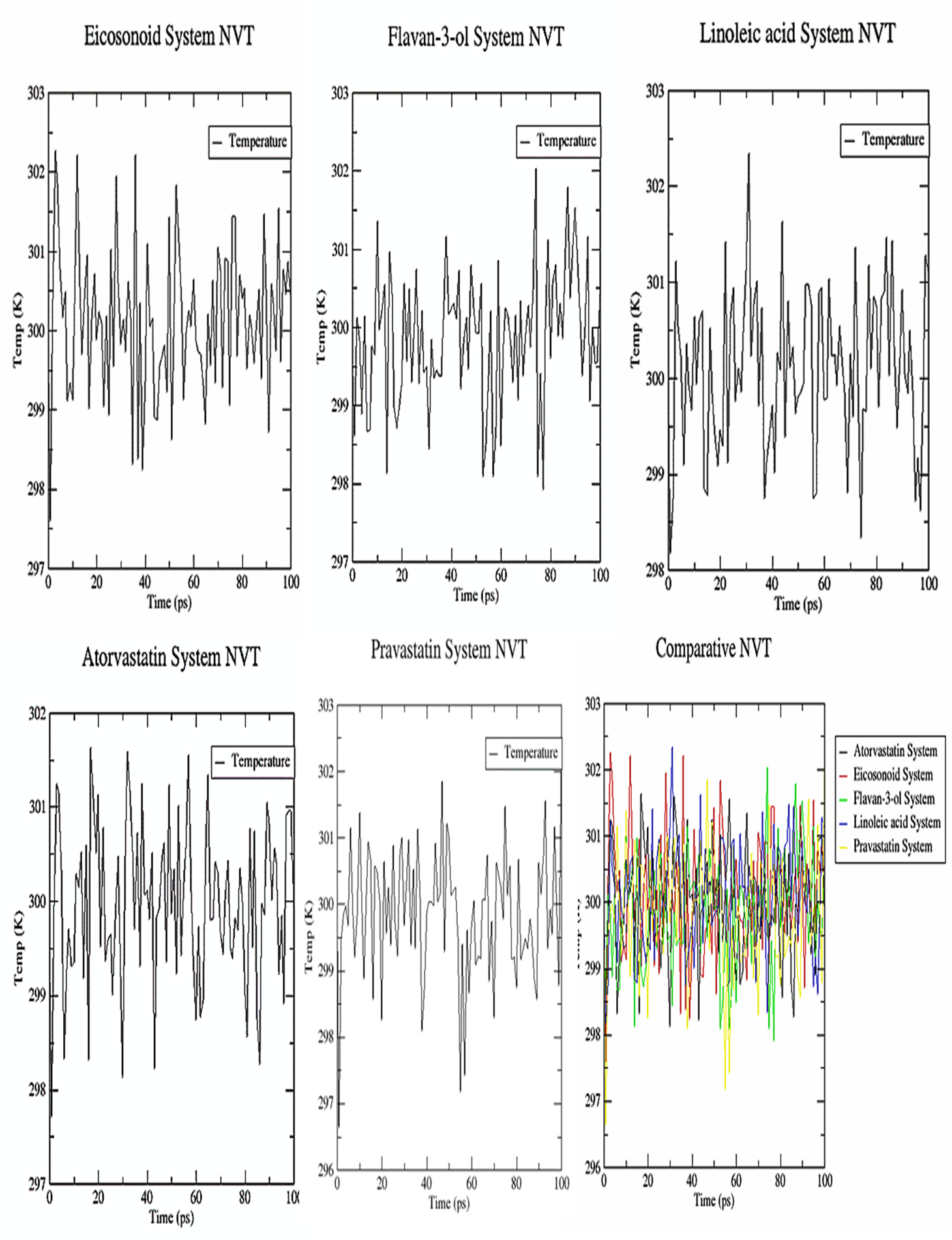


**S2A Fig:** System Temperature graphs of Eicosonoid, flavan-3-ol, linoleic acid, atorvastatin and pravastatin after temperature minimization for 100 picoseconds


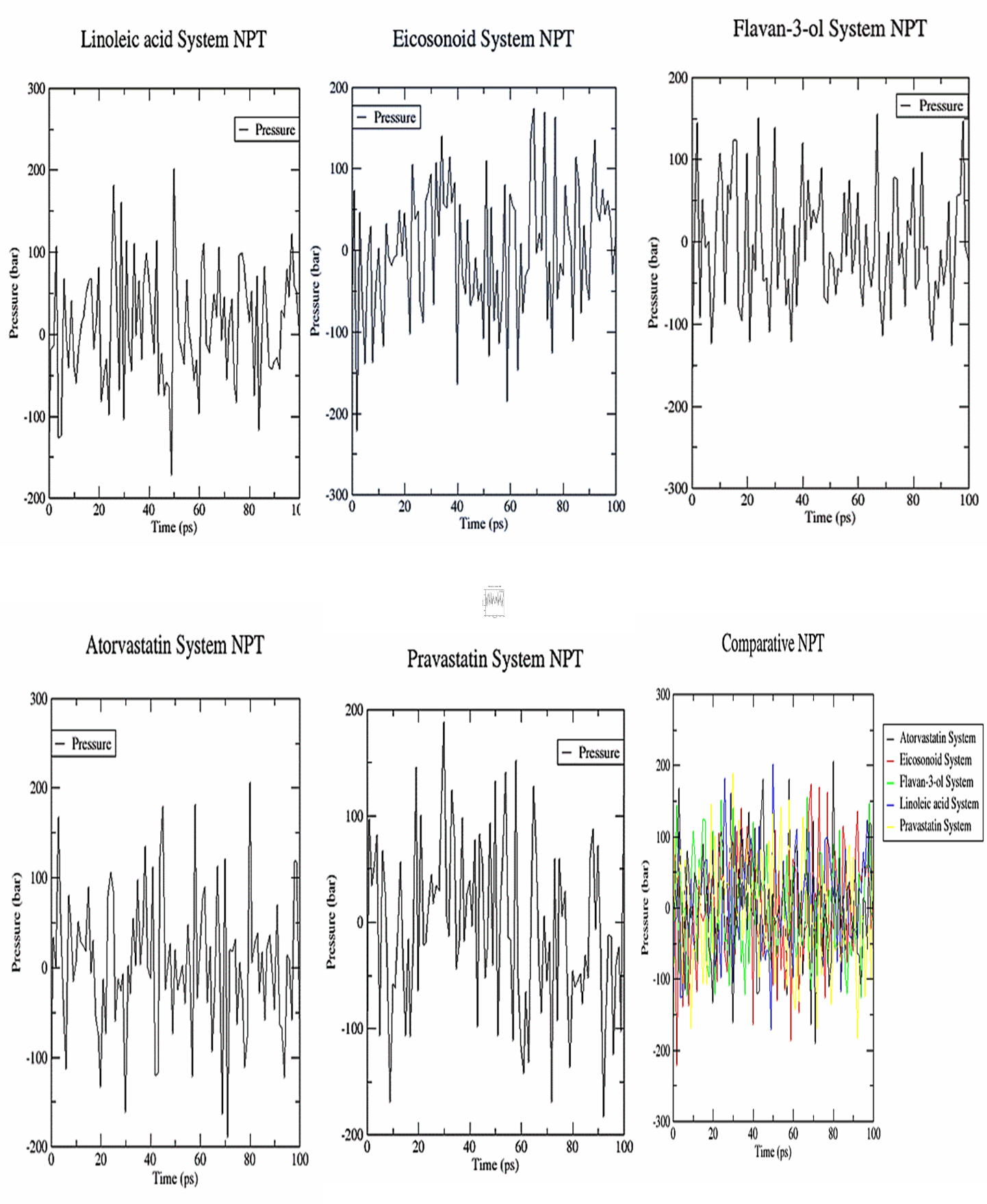


**S2B Fig:** System Pressure graphs of Eicosonoid, flavan-3-ol, atorvastatin, pravastatin and comparative view accounted after NPT Equilibration for 100ps


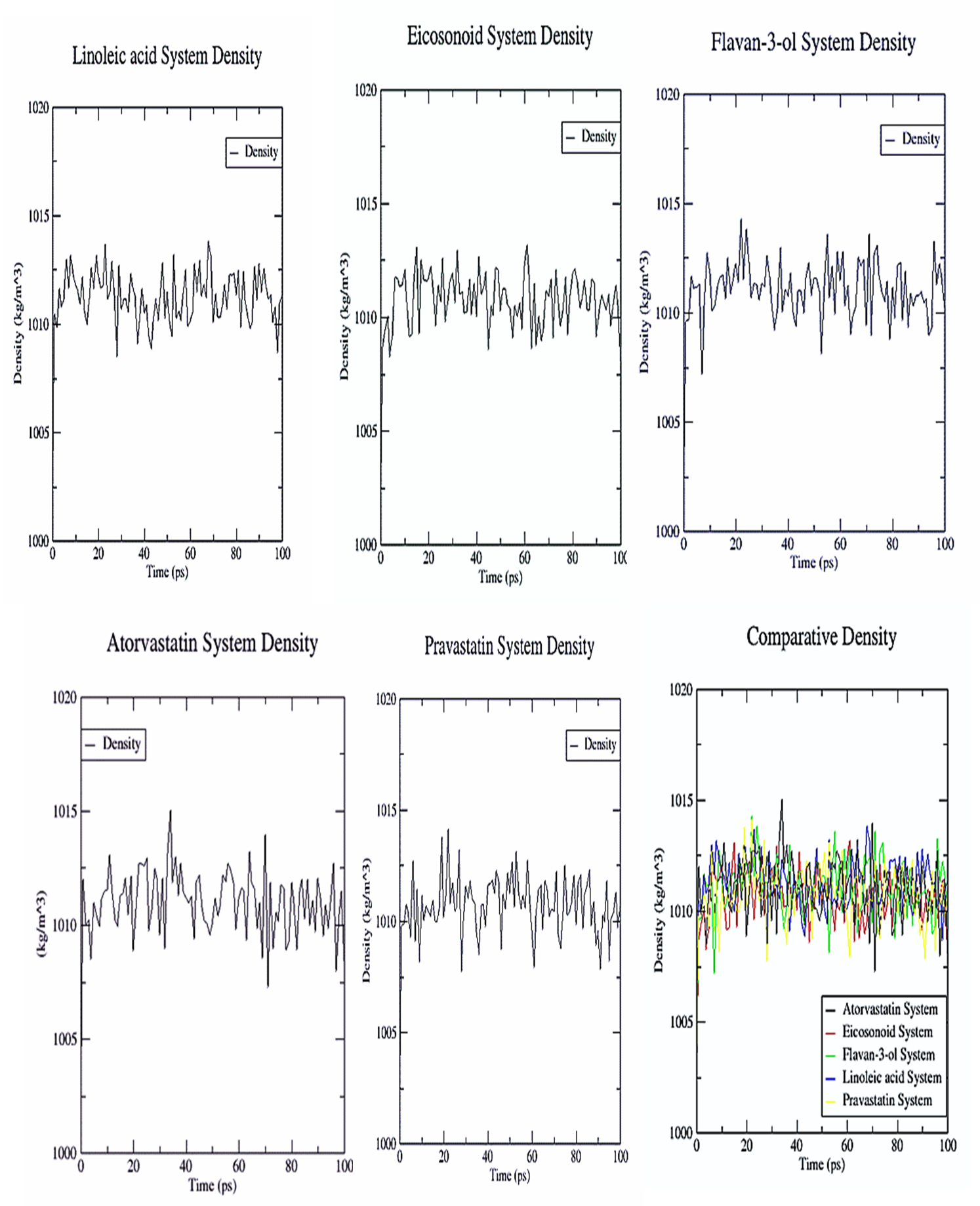


**S2C Fig:** System Density graphs of linoleic acid, Eicosonoid, flavan-3-ol, atorvastatin, pravastatin and comparative view accounted after Equilibration for 100ps


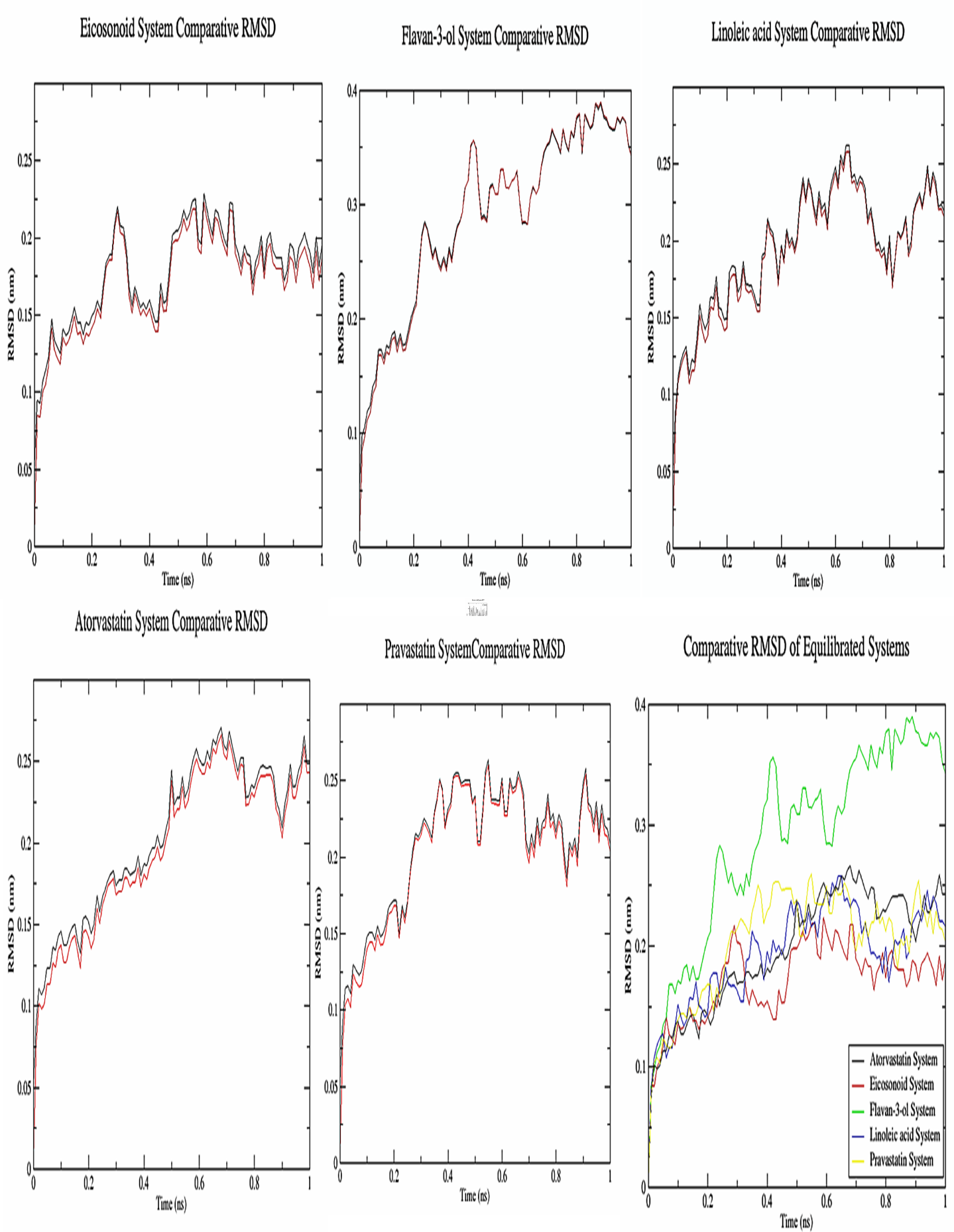


**S2D Fig:** System RMSD of Eicosonoid, Flavan-3-ol, linoleic acid, Pravastatin and atorvastatin. Crystal Backbone (black) Equilibrated Structure Backbone (red)


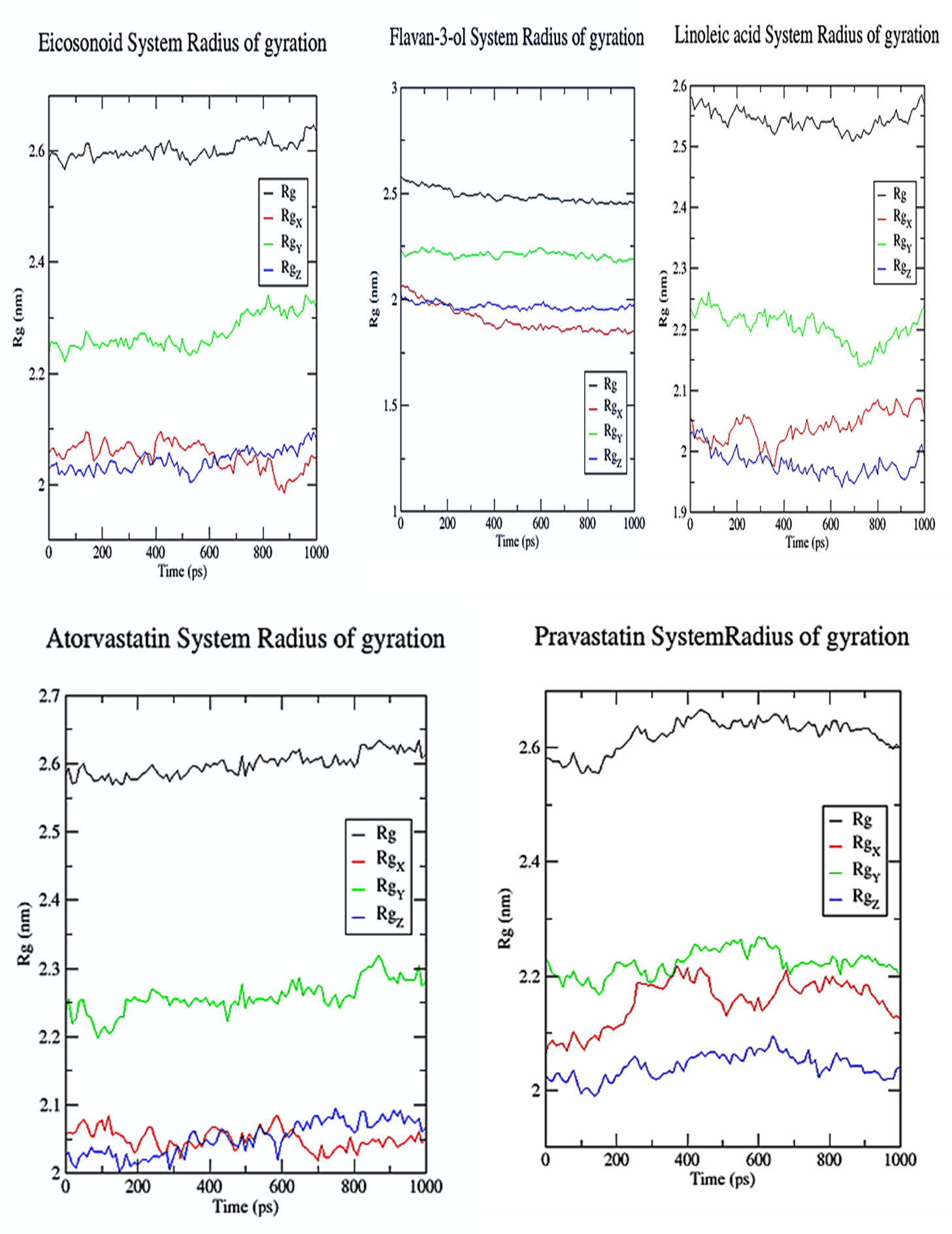


**S2E Fig:** Radius of gyration for Eicosonoid, Flavan-3-ol, linoleic acid, atorvastatin and pravastatin system accounted after 1000 ps.
