# Supplementary material for: Ameliorations in dyslipidemia and atherosclerotic plaque by the inhibition of HMG-CoA reductase and antioxidant potential of phytoconstituents of an aqueous seed extract of Acacia senegal (L.) Willd in rabbits
Source: PLoS One. 2022 Mar 3;17(3):e0264646. doi: 10.1371/journal.pone.0264646 (PMC8893677; doi:10.1371/journal.pone.0264646)
Supplement: Attachment — Submitted filename: Cover Letter-Point to Point responses -27.01.2022.pdf [file pone.0264646.s004.pdf]

Dr. H. Ram
Assistant Professor

Department of Zoology
Jai Narain Vyas University
Jodhpur (Raj.)-342005
Ph.: 09413033335 (M)
E-mail: hr.zo@jnvu.edu.in
baradhr@gmail.com
science.jnvu.edu.in/member_detail.php?mid=563&dept_id=28

Editor-In-Chief, Date: 28.01.2022

PLOS One

Subject: Submission of point-to-point responses against the comments.

Dear Sir/Madam,

I am submitting herewith point to point responses against the comments of submitted revised manuscript (PONE-D-21-34360) entitled "**Ameliorations in the biomarker indices of dyslipidemia and atherosclerotic plaque by the inhibition of HMG-CoA reductase and antioxidant potential of phytoconstituents of an aqueous seed extract of *Acacia senegal* (L.) Willd in rabbits.**" in prescribed format by incorporating your suggestions in your esteemed journal. I hope that the paper conforms to the rigorous standards of your journal, and I look forward to hearing your comments and decision concerning the publication of our paper. Please acknowledge and consider for publication.

Thanking you,

Yours Sincerely,

(Dr. Heera Ram)

Corresponding Author

Residence: IV/2, Sector -2, University Staff Colony, New Campus Road, Jodhpur (Raj), -342011, India

Dr. H. Ram
Assistant Professor

Department of Zoology
Jai Narain Vyas University
Jodhpur (Raj.)-342005
Ph.: 09413033335 (M)
E-mail: hr.zo@jnvu.edu.in
baradhr@gmail.com
science.jnvu.edu.in/member_detail.php?mid=563&dept_id=28

SN	Comments	Response
**1.**	Title need to short and modify.	Revised.
**2.**	Page11, Line106 need to modify.	Modify.
**3.**	Page 11 Line 114, typographic error, "--The inhibitory activity of increasing concentrations (0.32µg/ml, 0.62 µg/ml, 1.25 114 µg/ml, and 5µ0g/ml)…"	Revised.
**4.**	Methodology section should be updated by latest references.	Updated by latest references as per suggestions.
**5.**	Dyslipidemia indices should be explained properly and its roles in therapeutics of hypercholesterolemia.	Explanation incorporated.
**6.**	What are the independent and combined roles of dyslipidemia indices such as the Castelli indices (I & II), atherogenic coefficient and atherogenic risk indices?	There are indices of packing of free cholesterol and reverse cholesterol transport pathway.
**7.**	The molecular dynamics of interactions should be validated by SASA or structural analyses.	Incorporated.
**8.**	Discussion should be explained by the correlation between the antioxidant properties and hypocholesterolemic	Explanations incorporated.

Residence: IV/2, Sector -2, University Staff Colony, New Campus Road, Jodhpur (Raj), -342011, India

Dr. H. Ram
Assistant Professor

Department of Zoology
Jai Narain Vyas University
Jodhpur (Raj.)-342005
Ph.: 09413033335 (M)
E-mail: hr.zo@jnvu.edu.in
baradhr@gmail.com
science.jnvu.edu.in/member_detail.php?mid=563&dept_id=28

	activities of the phytocompounds.	
**9.**	Histopathology of aorta should be marked with presence of foam cells and regressed area.	Suggestion incorporated.
**10.**	Discussion should be revised with the explanations of molecular dynamics and molecular docking.	Discussion updated as per suggestions.
**11.**	Give the explanation of lipid peroxidation and hypercholesterolemia.	In hypercholesterolemia, there is an increase of total cholesterol pool in cells which results into altered cell membrane due to lipid peroxidation
**12.**	The druggability evidence should be updated and explained properly with latest references.	Updated and explanation incorporated.

(Dr. Heera Ram)
Corresponding Author
