# Supplementary material for: Ameliorations in dyslipidemia and atherosclerotic plaque by the inhibition of HMG-CoA reductase and antioxidant potential of phytoconstituents of an aqueous seed extract of Acacia senegal (L.) Willd in rabbits
Source: PLoS One. 2022 Mar 3;17(3):e0264646. doi: 10.1371/journal.pone.0264646 (PMC8893677; doi:10.1371/journal.pone.0264646)
Supplement: Attachment — Submitted filename: Comments.docx [file pone.0264646.s003.docx]

Ameliorations in the biomarker indices of dyslipidemia and atherosclerotic plaque by the inhibition of HMG-CoA reductase and antioxidant potential of phytoconstituents of an aqueous seed extract of *Acacia senegal* (L.) Willd in rabbits.

The manuscript is well organized and quiet interesting can be accepted after resolving the following minor queries.

1. Title need to short and modify.
2. Page11, Line106 need to modify.
3. Page 11 Line 114, typographic error, “--The inhibitory activity of increasing concentrations (0.32μg/ml, 0.62 μg/ml, 1.25 114 μg/ml, and 5μ0g/ml)…”
4. Methodology section should be updated by latest references.
5. Dyslipidemia indices should be explained properly and its roles in therapeutics of hypercholesterolemia.
6. What are the independent and combined roles of dyslipidemia indices such as the Castelli indices (I & II), atherogenic coefficient and atherogenic risk indices?
7. The molecular dynamics of interactions should be validated by SASA or structural analyses.
8. Discussion should be explained by the correlation between the antioxidant properties and hypocholesterolemic activities of the phytocompounds.
9. Histopathology of aorta should be marked with presence of foam cells and regressed area.
10. Discussion should be revised with the explanations of molecular dynamics and molecular docking.
11. Give the explanation of lipid peroxidation and hypercholesterolemia.
12. The druggability evidence should be updated and explained properly with latest references.
